# Crown Group Lejeuneaceae and Pleurocarpous Mosses in Early Eocene (Ypresian) Indian Amber

**DOI:** 10.1371/journal.pone.0156301

**Published:** 2016-05-31

**Authors:** Jochen Heinrichs, Armin Scheben, Julia Bechteler, Gaik Ee Lee, Alfons Schäfer-Verwimp, Lars Hedenäs, Hukam Singh, Tamás Pócs, Paul C. Nascimbene, Denilson F. Peralta, Matt Renner, Alexander R. Schmidt

**Affiliations:** 1 Department of Biology and Geobio-Center, University of Munich (LMU), Munich, Germany; 2 School of Marine and Environmental Sciences, University of Malaysia Terengganu, Terengganu, Malaysia; 3 Mittlere Letten 11, Herdwangen-Schönach, Germany; 4 Swedish Museum of Natural History, Stockholm, Sweden; 5 Birbal Sahni Institute of Palaeobotany, Lucknow, India; 6 Botany Department, Eszterházy University, Eger, Hungary; 7 Division of Invertebrate Zoology, American Museum of Natural History, New York, New York, United States of America; 8 Instituto de Botânica, São Paulo, Brazil; 9 Royal Botanic Gardens and Domain Trust, Sydney, Australia; 10 Department of Geobiology, University of Göttingen, Göttingen, Germany; Institute of Botany, CHINA

## Abstract

Cambay amber originates from the warmest period of the Eocene, which is also well known for the appearance of early angiosperm-dominated megathermal forests. The humid climate of these forests may have triggered the evolution of epiphytic lineages of bryophytes; however, early Eocene fossils of bryophytes are rare. Here, we present evidence for lejeuneoid liverworts and pleurocarpous mosses in Cambay amber. The preserved morphology of the moss fossil is inconclusive for a detailed taxonomic treatment. The liverwort fossil is, however, distinctive; its zig-zagged stems, suberect complicate-bilobed leaves, large leaf lobules, and small, deeply bifid underleaves suggest a member of Lejeuneaceae subtribe Lejeuneinae (*Harpalejeunea*, *Lejeunea*, *Microlejeunea*). We tested alternative classification possibilities by conducting divergence time estimates based on DNA sequence variation of Lejeuneinae using the age of the fossil for corresponding age constraints. Consideration of the fossil as a stem group member of *Microlejeunea* or *Lejeunea* resulted in an Eocene to Late Cretaceous age of the Lejeuneinae crown group. This reconstruction is in good accordance with published divergence time estimates generated without the newly presented fossil evidence. Balancing available evidence, we describe the liverwort fossil as the extinct species *Microlejeunea nyiahae*, representing the oldest crown group fossil of Lejeuneaceae.

## Introduction

Bryophytes (liverworts, mosses and hornworts) likely played a major role in the early Paleozoic colonization of terrestrial ecosystems by plants [1–3]. However, reconstruction of the early evolution of plants on land is hampered by the meagre fossil record [4–5] as well as deviating hypotheses on the relationships of the main land plant lineages [6–7] and their time of origin [8–10].

Traditionally, bryophytes have been considered as “unchanging, unmoving sphinxes of the past” [11] whose ranges were largely shaped by vicariance and extinction events [12–13]. Following this assumption some extant species were considered to be Jurassic in age [14]. This view was recently challenged by molecular phylogenetic evidence indicating Cretaceous or Cenozoic ages of many extant bryophyte genera [15–20] and diversification rate estimates for the most recently derived lineages that are comparable to those of angiosperms [21]. Nevertheless, estimated ages for some nodes in the different studies vary considerably under influence of assumed maximum ages of lineages, standard mutation rates, and deviating methods of node calibration [17,20,22–26]. To resolve robust divergence time estimates and better understand bryophyte evolution we need to improve our knowledge of the fossil record and explore promising new fossil deposits.

Paleozoic and early Mesozoic bryophyte fossils are scarce [15,27–28] and the evaluation of these early fossils is difficult since only a few late Mesozoic fossils are preserved in cellular detail. Thus, their classification usually causes great difficulties. *Diettertia montanensis*, for example, was initially described as a moss but subsequently treated as a jungermannealean liverwort [29]. Many Cenozoic mosses and leafy liverworts are exquisitely preserved as amber inclusions [30–31]. Amber, fossilized tree resin of gymnosperms and angiosperms, is well known for its numerous botanical, zoological and fungal inclusions. Although the morphology of bryophyte amber fossils usually closely resembles that of extant genera, the extent of morphological information that can be collected from fossils rarely compares to that obtainable from extant species. Accordingly, their taxonomic interpretation is a challenging task and subject to a certain degree of uncertainty [32].

Leafy liverworts are split into two main lineages: the generalist Jungermanniales and the largely epiphytic Porellales [33]. Porellales’ habitat preference make them prime candidates for becoming encased in resin flows; indeed most amber fossils of liverworts belong to this order. Their largest family is the Lejeuneaceae, representing a derived lineage with more than 1,000 extant species in some 70 currently accepted genera, with a center of diversity in the humid tropics [34–36]. Numerous Lejeuneaceae fossils have been found in Miocene Dominican and Mexican amber, in addition to several inclusions in Paleogene Baltic, Bitterfeld and Rovno amber [31].

Mosses include a speciose derived lineage characterized by its predominantly creeping or pendant growth, tapered “prosenchymatous” leaf cells and sporophytes on short lateral branches. Like Porellales, they include numerous epiphytes and are frequently embedded in amber, yet many fossils do not show the character states necessary for a reliable identification [30,37].

Only recently, early Eocene (Ypresian) Cambay amber [38] was discovered and determined to be a promising fossil deposit. This Indian amber has already yielded numerous zoological inclusions as well as inclusions of fungi and remains of the resin-producing Dipterocarpaceae [39–44]. Here, we present the first bryophytes from Indian amber. We discuss the taxonomic relationships of a lejeuneoid liverwort using both morphological evidence and divergence time estimates based on molecular evidence. We describe the extinct species *Microlejeunea nyiahae*, and also a pleurocarpous moss with unclear taxonomic relationships.

## Material and Methods

### Amber fossils

Amber piece AMNH-Tad-441-A was found in the Tadkeshwar Lignite Mine of Gujarat State, western India (N 21° 21.400, E 073° 04.532), which contains outcrops of early Eocene (Ypresian, 52 million year-old [45]) shallow marine sediments. This amber originates from trees of the Dipterocarpaceae that grew in a fully tropical environment [40]. After an initial inspection of the inclusions, followed by preliminary polishing of amber surfaces, the specimen was embedded in a high-grade epoxy resin [EPO-TEK 301–2, Epoxy Technology Inc., mixing ratio 100 (resin): 35 (hardener) by weight] in a procedure modified from the protocols described by Nascimbene and Silverstein [46]. After curing, the sample was trimmed and polished on opposite sides using a series of wet silicon carbide abrasive papers (Struers, Germany) with decreasing grit sizes [grit from FEPA P 600–4000 (25.8 μm to 5 μm particle size)]. The piece of amber is currently housed in the amber collection of the Division of Invertebrate Zoology of the American Museum of Natural History (AMNH), New York, USA. It will ultimately be deposited in the amber collection of the Birbal Sahni Institute of Palaeobotany, Lucknow, India. The specimen is at all times publicly deposited and accessible.

**Fig 1:**
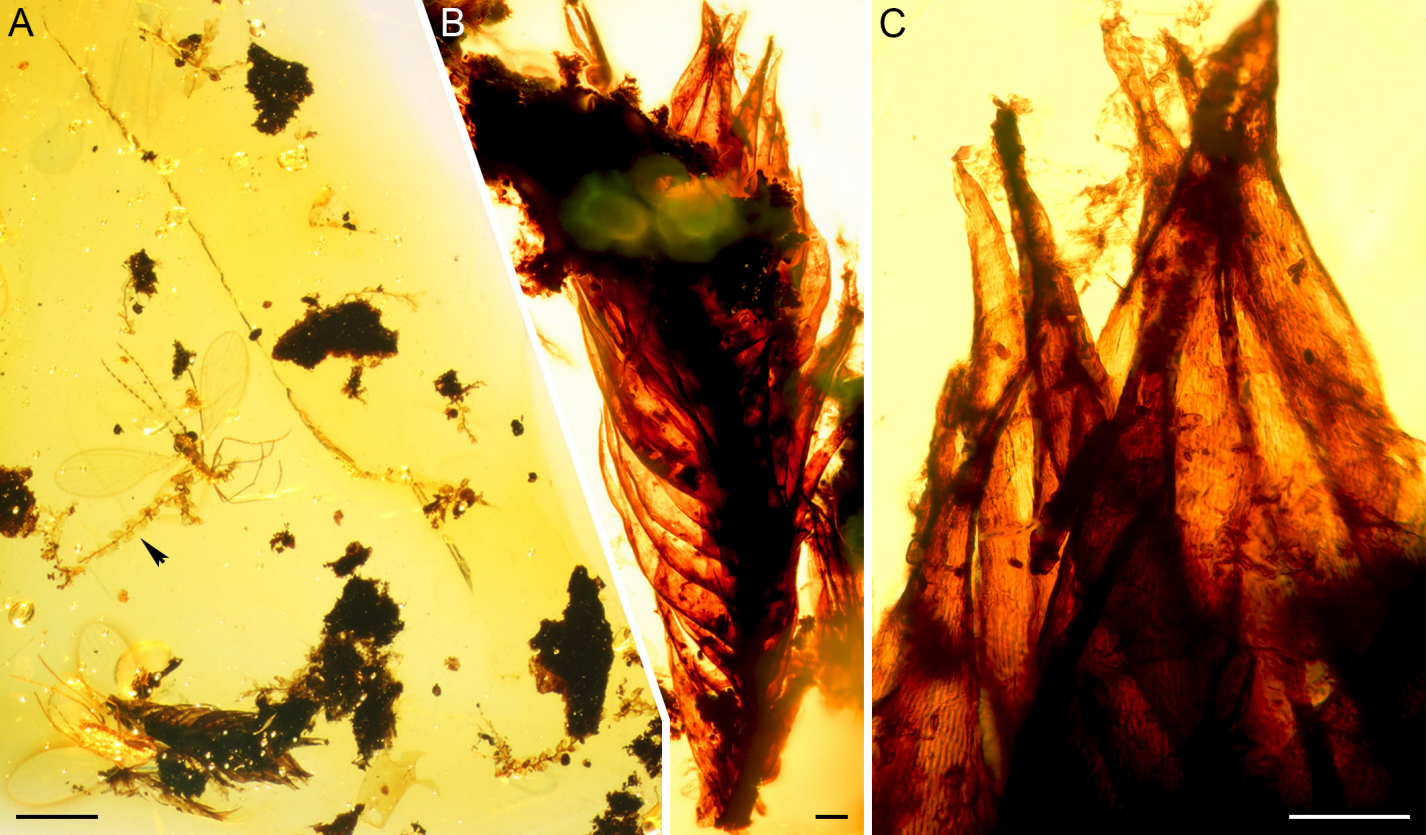
Cambay amber specimen AMNH-Tad-441-A. (A) Overview showing liverwort and moss inclusions as well as two dipterans. The arrowhead points to the holotype of *Microlejeunea nyiahae*. (B) Pleurocarpous moss. (C) Close-up showing upper portions of leaves of the moss inclusion. The prosenchymatous cells are well visible. Scale bars 1 mm (A) and 100 μm (B,C).

**Fig 2:**
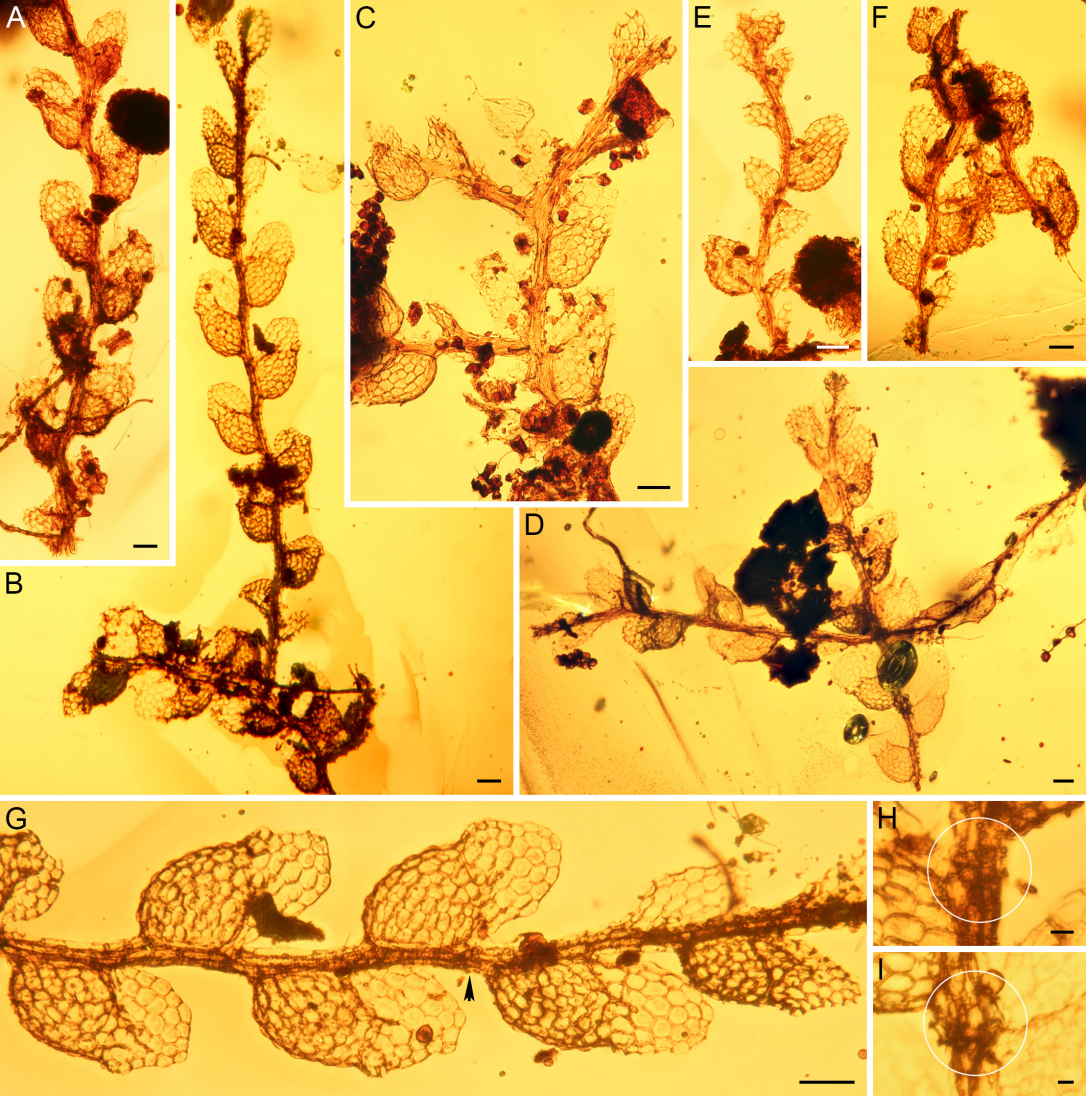
*Microlejeunea nyiahae* sp. nov. (AMNH-Tad-441-A) from Eocene Cambay amber. (A-F) Gametophytes; (G) Portion of the shoot depicted in (B); the arrowhead points to the underleaf that is enlarged in (H). (H, I) Deeply bifid underleaves (encircled). The gametophyte fragment shown in B and G represents the holotype. Scale bars 50 μm (A-G) and 10 μm (H,I).

The specimen was studied using a dissection microscope (Carl Zeiss Stemi 2000) and a compound microscope (Carl Zeiss AxioScope A1), equipped with Canon 60D digital cameras. In some instances, incident and transmitted light were used simultaneously. The images of Figs 1 and 2 are digitally stacked photomicrographic composites of 10 to 45 individual focal planes obtained using the software package HeliconFocus 6.5. Several fragments of a leafy liverwort matching the morphology of Lejeuneaceae subtribe Lejeuneinae, as well as a branch of a moss, two dipterans and a springtail are enclosed in this amber piece. The taxonomic treatment of the bryophyte inclusions is based on literature data on fossil and extant bryophytes, and on comparisons with herbarium specimens housed at the herbarium Eger (EGR), the Göttingen University Herbarium (GOET), the Bavarian State Collection of Botany (M), the Swedish Museum of Natural History in Stockholm (S), the Herbarium São Paulo (SP), and the Royal Botanical Garden Sydney (NSW).

No permits were required for the described study, which complied with all relevant regulations.

### Divergence time estimates

The morphology of the liverwort fossil (see Results) suggests an affiliation to *Microlejeunea* or the extant *Lejeunea exilis* [47–48] of Lejeuneaceae subtribe Lejeuneinae. This subtribe comprises the genera *Harpalejeunea*, *Microlejeunea* and *Lejeunea* [36,49–50]. In earlier studies, the Lejeuneinae crown group was estimated to have an Oligocene [51] or Paleocene age [18]. We tested various possible taxonomic treatments of the liverwort by conducting divergence time estimates based on a DNA sequence alignment of Lejeuneinae using the age of the liverwort fossil for corresponding age constraints. The resulting phylogenetic chronograms were compared with the published divergence time estimates generated without the newly presented fossil evidence. We assembled a three marker alignment of Lejeuneinae (nrITS, cp *rbc*L and *trn*L-*trn*F) based on the comprehensive sampling of Heinrichs et al. [52] using one accession per species and favoring accessions for which all three markers were available. Accessions of *Lepidolejeunea* were chosen as outgroup based on the phylogenetic hypotheses of Wilson et al. and Bechteler et al. [34,36]. Based on our current understanding of global species diversity, we sampled *Harpalejeunea*, *Lejeunea*, and *Microlejeunea* proportionally to represent about 10% of the extant species diversity. To arrive at a balanced sampling, we used not only GenBank sequences but sequenced additional accessions of *Harpalejeunea* and *Microlejeunea* using the protocols and sequencing facilities described in [36] (S1 Table).

Dating relied on the BEAST package 1.8.2 [53] and the TIM3+Γ+I substitution model for the ITS partition, the TVM+Γ substitution model for *trn*L-F, and GTR+Γ+I for *rbc*L as selected by jmodeltest under the AIC criterion [54–55], with four gamma categories. All parameters were estimated in BEAST. The tree prior was a pure birth (Yule) tree and MCMC was run for 50 million generations, sampling every 5,000 generations. Convergence was determined by examining the log files in Tracer 1.6. ESS values > 200 indicated that the parameter space had been sampled sufficiently for valid parameter estimation. To find the appropriate clock model, a likelihood ratio test [56] was carried out in PAUP* 4.0a146 [57]. A strict clock was rejected (*P < 0.05), and thus an uncorrelated lognormal relaxed clock model was employed [58]. Five different divergence time estimates were conducted using the 52 million-year-old Lejeuneinae amber fossil for different age constraints, and using a normal distribution prior with a standard deviation of 5 Ma. The fossil was assigned either to *Microlejeunea* or to *Lejeunea*, first as most recent common ancestor, and secondly using the “include stem” option. Lastly, the fossil was assigned to the branch of the extant *Lejeunea exilis*.

### Nomenclature

The electronic version of this article in a Portable Document Format (PDF) in a work with an ISSN or ISBN will represent a published work according to the International Code of Nomenclature for algae, fungi, and plants, and hence the new names contained in the electronic publication of a PLOS ONE article are effectively published under that Code from the electronic edition alone, so there is no longer any need to provide printed copies. The online version of this work is archived and available from the following digital repositories: PubMed Central, LOCKS.

## Results

### Gametophyte fragments of a Lejeuneaceae representative

**Fig 3:**
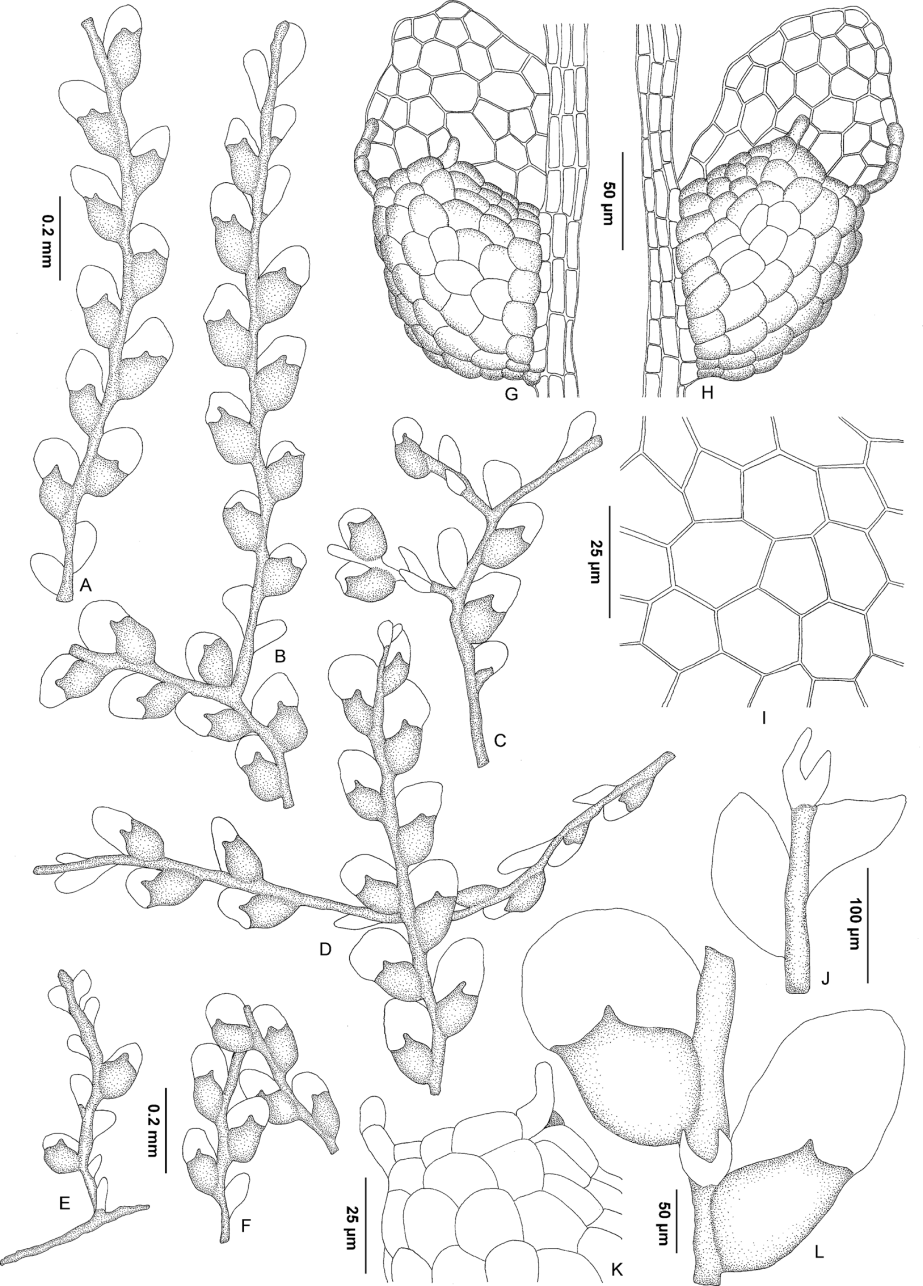
Reconstruction of *Microlejeunea nyiahae* based on the holotype and accompanying gametophytes. (A-F) Portions of sterile shoots in ventral view. (G-H) Portion of stem with a leaf in ventral view showing the large leaf lobule (dotted cells) and the lobe. (I) Median leaf lobe cells in top view. (J, L) Portion of shoot in ventral view with deeply bifid underleaf. (K) Free margin of leaf lobule showing hyaline papilla cell (gray) next to apical lobule tooth.

*Microlejeunea nyiahae* Heinrichs, G.E.Lee, Schäf.-Verw. & A.R.Schmidt, sp. nov. (Figs 2 and 3)

#### Holotype

American Museum of Natural History, AMNH-Tad-441-A, Fig 2B and 2G show the gametophyte fragment representing the holotype; its location in the amber piece is indicated by the arrowhead in Fig 1A.

#### Etymology

The specific epithet honors Nyiah Goff (Coatesville, Indiana) who discovered the bryophyte fossils described in this study.

#### Description

Plants sterile, 0.2–0.3 mm wide, irregularly and infrequently branched, lateral branches spreading and usually few, 0.25–1.50 mm in length. Stem straight to shallowly zig-zagged, 24–30 μm in diameter, with a 2-cells wide ventral merophyte. Leaves incubous, plane and distant, sometimes contiguous. Leaf lobes 0.15–0.20 mm long, 0.06–0.10 mm wide, ovate-oblong to triangularly ovate; leaf apex broadly rounded, rarely subtruncate, flat; leaf margins entire; ventral margin forming an angle of 150°-180° with keel; free dorsal margin hardly more than reaching stem. Leaf cells rather uniform, pentagonal to hexagonal, irregularly quadrate to rectangular towards leaf margin; apical cells 18–20 μm long and 15–17 μm wide, median and basal cells 18–25 μm long and 15–20 μm wide; cell walls hyaline, with small or indistinct trigones and without intermediate thickenings; cell surfaces smooth. Leaf lobules sometimes reduced at the base of stem or branches and at apex of shoots, 0.10–0.15 mm long, ca. 0.10 mm wide, to 1/2-2/3 length of lobe, at an angle of 60°-80° to stem, ovate to suboblong, inflated along keel; apex obliquely truncate; keel curved, crenate; free margin incurved; first lobule tooth often collapsed, 15–17 μm long, oblong, sometimes deflexed, apex obtuse; large disc cell (cell below the first tooth) present, 20–22 μm long and 10–12 μm wide, hyaline papilla inserted on lobule margin at base of first tooth. Underleaves small, 0.04–0.06 mm long, 0.04–0.05 mm wide, to 1.5 times wider than stem, distant, ovate; bilobed, lobes 1/2-2/3 of underleaf length, about 2 cells wide, oblong to lanceolate, distant; sinus narrow to broad, acute, V-shaped; tips acute to obtuse; underleaf margin entire; base ± cuneate, insertion line straight. Rhizoids not seen.

### Gametophyte fragment of a pleurocarpous moss

Sterile branch with ventral leaves more or less erect (Fig 1B and 1C), dorsal leaves from patent base curved forward. Leaves narrowly ovate or lanceolate, 0.90–1.00 mm long (width not possible to measure accurately), gradually narrowed upwards to an acuminate apex, concave, with channelled to semi-tubular acumen; costa lacking or not visible; margin plane, entire or almost so. Median lamina cells linear, 4.5–6.0 μm wide, ca. 75 μm long, thin-walled. Differentiated alar cells obviously present.

### Divergence time estimates

**Fig 4:**
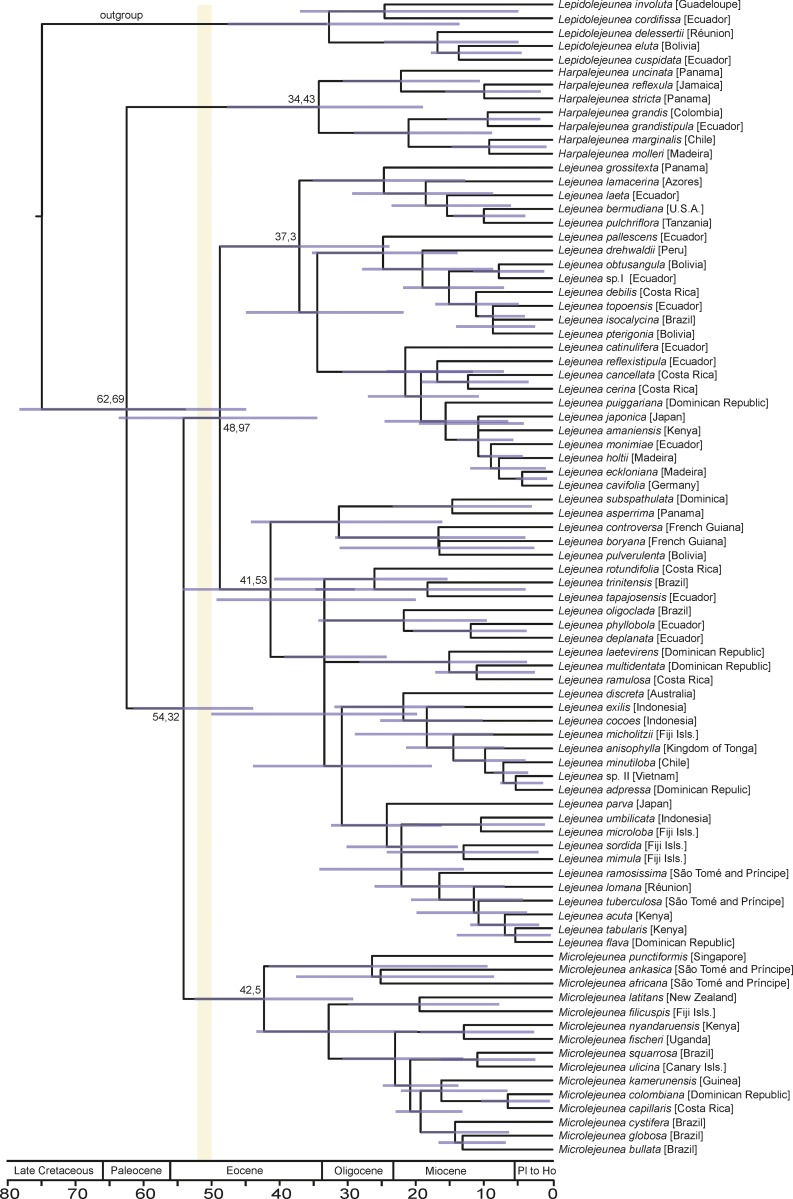
Phylogenetic chronogram for Lejeuneaceae subtribe Lejeuneinae considering the fossil as a stem lineage element of *Microlejeunea*. Time scale shown in million years to present (Pl to Ho = Pliocene to Holocene). Confidence age estimates shown as horizontal bars. Vertical bar indicates time interval for Cambay amber. Amber from the Tadkeshwar Lignite Mine has an age of 52 Ma.

The results of the divergence time estimates are presented in Table 1. Assignment of the lejeuneoid fossil to the *Lejeunea exilis* node leads to an Early Cretaceous to Middle Triassic age reconstruction of the Lejeuneinae crown group [170 Ma, confidence interval 101–239 Ma]. Assignments to the *Lejeunea* stem lineage or to the *Microlejeunea* stem lineage (Fig 4) yield an Eocene to Late Cretaceous age of this crown group [*Lejeunea* assignment: 57 Ma, confidence interval 39–74 Ma; *Microlejeunea* assignment: 63 Ma, confidence interval 45–78 Ma].

**Table 1:** Results from the different divergence time estimates of Lejeuneinae. Age estimates are given as mean plus 95% confidence interval.

Fossil calibration node	Node age [95% HPD interval]			
	*Microlejeunea-Lejeunea*-*Harpalejeunea*	*Microlejeunea-Lejeunea*	*Microlejeunea*	*Lejeunea*
*Lejeunea exilis*	169.55 [100.9;238.62]	161.81 [114.03;204.05]	110.07 [57.83;156.18]	133.26 [91.38;170.70]
*Lejeunea*	65.84 [45.89;88.74]	62.31 [48.05;74.00]	42.8 [26.73;48.2]	52.21 [42.1;60.12]
*Lejeunea* stem	56.86 [39.17;74.23]	52.21 [42.08;59.55]	37.05 [21.4;48.28]	45.13 [33.66;54.21]
*Microlejeunea*	79.80 [54.28;101.41]	70 [52.61;82.9]	53.64 [41.72;60.5]	62.19 [41.63;79.82]
*Microlejeunea* stem	62.69 [45.09;78.45]	54.32 [44.07;61.68]	42.49 [29.36;52.72]	48.97 [34.65;63.84]

## Discussion

### Liverwort inclusions

Liverworts of the family Lejeuneaceae are very abundant in the humid tropics, making up a large part of the local epiphytic liverwort diversity [34,59]. With some 1,000 to 1,500 species in about 70 genera, they are the largest family of liverworts [35–36,60–63]. They are not only common in contemporary tropical rain forests but also have a comprehensive fossil record [31,64]. The oldest putative Lejeuneaceae fossil is the poorly preserved Middle Jurassic compression fossil *Sinolejeunea yimaensis* [65]. Although its position in Lejeuneaceae is weakly supported considering the presented evidence; it is in accordance with the recently inferred late Triassic origin of Lejeuneaceae by Feldberg et al. [18], who did not use this fossil as an age constraint. Previously identified Lejeuneaceae fossils occur in Paleogene Baltic, Bitterfeld, and Rovno amber as well as in Miocene Dominican and Mexican amber [31]. Although their generic placement is sometimes subject to controversy [66–67], there is not much doubt about their affiliation to crown group clades of Lejeuneaceae [51]. Identification of the precise age of the Paleogene fossils is, however, sometimes problematic. The Eocene sediments that yield the majority of Baltic amber are 35–47 million years old, but some amber is also found in up to 50 million-year-old strata [68–69]. Ukrainian Rovno amber is considered to have roughly the same age as Baltic amber [70–71] but detailed information is not yet available. Bitterfeld amber originates from the open brown coal pit Goitzsche near the city of Bitterfeld in central Germany. The amber-bearing sediment is uppermost Oligocene (24 Ma) in age [72–73] but some authors suggest that Bitterfeld amber has been re-deposited and that it is contemporaneous with Baltic amber [74]. In contrast, the proposed early Eocene (50–52 Ma) age of Indian Cambay amber [40] is much more precisely and reliably established; thus, its biological inclusions are better suited to calibrate phylogenetic trees. Cambay amber originates from the warmest period of the Eocene, which is also well known for the occurrence of early angiosperm-dominated megathermal forests [75–76]. Indeed, Cambay amber was produced by trees of the angiosperm family Dipterocarpaceae, which are common in extant tropical lowland rain forests [39–41]. Cambay amber thus provides an opportunity to study epiphytic bryophytes from early Eocene angiosperm forests, lineages that benefitted from the more humid microclimate of these forests compared to Cretaceous gymnosperm forests [18,21,77–79].

Present-day Asian rainforests are rich in epiphytic Lejeuneaceae [80,81]; hence the occurrence of representatives of this family in Cambay amber is not unexpected. The liverwort inclusions in the investigated amber piece share a consistent morphology and are thus considered to belong to a single species (Figs 2 and 3). They comprise several delicate gametophytes with zig-zagged stems, suberect leaves with large lobules and rounded lobes, unicellular first lobule teeth, marginal hyaline papillae, small, deeply bifid underleaves with a V-shaped sinus and forward-directed, acute lobes. This combination of character states indicates that the fossil belongs to a representative of Lejeuneaceae subtribe Lejeuneinae, either being a member of *Microlejeunea* or of *Lejeunea*. *Harpalejeunea* has underleaves with a wide, U-shaped sinus and broadly rounded lobes, and acute to acuminate leaf lobes whose long axis is orientated perpendicular to the stem [35–36,49–50]. *Microlejeunea* has a more consistent morphology than *Lejeunea* and can be separated by winged female bracts and the presence of ocelli in at least some leaves [49]. Ocelli are specialized cells containing only a single large oil body [82]; however, oil bodies are usually not preserved in fossils. Since the ocelli of *Microlejeunea* have the same size as the surrounding leaf cells, it is not possible to confirm their presence or absence in the investigated fossil [see 64]. While female bracts are also not preserved, the forward pointed leaves and the large leaf lobules of the sterile gametophytes fully match the morphology of extant *Microlejeunea* [83–85]. Representatives of *Lejeunea* usually have straight stems, more prominent leaf lobes and larger underleaves but several extant taxa resemble *Microlejeunea* in vegetative characters. Especially weak shoots and branches of the widespread Asian-Malesian *Lejeunea exilis* resemble the fossil; however, well developed shoot systems of *Lejeunea exilis* have elongate triangular, acute lobes [47–48]. *Lejeunea exilis* and other Palaeotropical species of *Lejeunea* are placed in derived lineages in the most comprehensively sampled Lejeuneinae phylogeny available to date [52]. Constraining the *Lejeunea exilis* clade of our phylogeny with the fossil’s age yielded an Early Cretaceous or Jurassic age of *Lejeunea* (Table 1). Published divergence time estimates suggest an Oligocene or Eocene origin of *Lejeunea* [18,51]; hence we consider a relationship of the fossil to the extant *Lejeunea exilis* unlikely. Morphological similarity between the fossil and *L*. *exilis* is probably the result of convergence. Evidence for morphological convergence between fossil and extant species was strongly supported in a study of *Radula*, another lineage within the Porellales [86]. The assumption of an early crown group or stem group member of *Microlejeunea* leads to estimates of a Paleogene origin of *Lejeunea* and *Microlejeunea* and is thus in better accordance with published chronograms. The relationships of the three Lejeuneinae genera *Lejeunea*, *Harpalejeunea* and *Microlejeunea* are not yet fully resolved [49], but *Lejeunea* and *Microlejeunea* form a sister relationship in our chronograms. Alternative assignment of *Microlejeunea nyiahae* to the *Lejeunea* or *Microlejeunea* lineage thus results in largely similar divergence time estimates. Accordingly, our taxonomic decision does not cause misleading divergence time estimates, even if the fossil belonged to *Lejeunea* rather than *Microlejeunea*. A further argument in favor of *Microlejeunea* is the presence of Palaeotropic accessions in early diverging lineages (Fig 4), whereas the early diverging lineages of *Lejeunea* are Neotropical [52]. This hypothesis needs to be tested using an extended taxon sampling since our current sampling includes only some 10% of the extant diversity. So far, *Microlejeunea* and *Lejeunea* fossils have only been found in Miocene amber from the Dominican Republic [64,87]; the Miocene Mexican amber inclusion *Lejeunea palaeomexicana* has recently been transferred to *Ceratolejeunea* [67].

Assumption of a stem group representative of *Microlejeunea* is in accordance with published divergence time estimates that do not rely on the new fossil from Cambay amber; yet our reconstruction (Fig 4) supports older [18] rather than younger [51] age estimates for Lejeuneaceae. The presented evidence leads to the conclusion that the genera of subtribe Lejeuneinae were established in the Late Cretaceous or Paleogene. This scenario supports a crown group diversification of Lejeuneaceae genera in Cenozoic, angiosperm-dominated forests [18,21].

### Pleurocarpous moss

The linear, prosenchymatous cells of the second bryophyte species in the investigated piece of amber are indicative of a pleurocarpous moss (Fig 1B and 1C), but the available morphological information is inconclusive for a reliable classification at the level of family or below. Alar cells, groups of differentiated cells in the basalmost regions of the leaf, are of prime importance for the identification of supraspecific taxa of pleurocarps. The arrangement of the basal leaf cells points to the presence of alar cell groups, but the alar cells themselves have been lost. Hence, we abstain from a formal description while reporting the first moss in Cambay amber. Pleurocarpous mosses occur in many habitats and are abundant as epiphytes, especially in tropical areas [88–89]. Divergence time estimates indicate their presence since the Cretaceous [90].

### Perspectives

Indian Cambay amber is a promising deposit not only for zoological inclusions but also for plant fossils. The early Eocene resin has preserved remains of Dipterocarpaceae forest ecosystems from the Early Eocene Climatic Optimum (EECO). This period is possibly of prime importance for the establishment of epiphytic plant lineages [77–78,91]; hence Cambay fossils have a significant impact on improving our knowledge of the evolution of angiosperm-dominated tropical forest ecosystems and on the influence of the rise of angiosperms on epiphyte diversity.

## Supporting Information

S1 Table Taxa used in the present study. (PDF)
