# Supplementary material for: Crown Group Lejeuneaceae and Pleurocarpous Mosses in Early Eocene (Ypresian) Indian Amber
Source: PLoS One. 2016 May 31;11(5):e0156301. doi: 10.1371/journal.pone.0156301 (PMC4887038; doi:10.1371/journal.pone.0156301)
Supplement: S1 Table — (PDF) [file pone.0156301.s001.pdf]

S1 Table. Taxa used in the present study. Origin of the studied material, vouchers, and GenBank accession numbers. New sequences are indicated in bold face.

**Taxon**	**Voucher**	***rbc*L**	***trn*L-F**	**nrITS**
Harpalejeunea grandis Grolle & M.E.Reiner	Colombia, Cleef 6450 (Paratype, GOET)	KC313144	KC313184	KC313106
Harpalejeunea grandistipula R.M.Schust.	Ecuador, Schäfer-Verwimp 24289	DQ983685	DQ987451	DQ987351
Harpalejeunea marginalis (Hook. f. & Taylor) Steph.	Chile, Schäfer-Verwimp & Verwimp 8095 (GOET)	**KX113504**	**KX113480**	**KX113492**
Harpalejeunea molleri (Steph.) Grolle	Madeira, Schäfer-Verwimp & Verwimp 25732/B (GOET)	**KX113505**	**KX113481**	**KX113493**
Harpalejeunea reflexula A.Evans	Jamaica, Schäfer-Verwimp 35089 (GOET)	**KX113506**	**KX113482**	**KX113494**
Harpalejeunea stricta (Lindenb. & Gottsche) Steph.	Panama, Schäfer-Verwimp & Verwimp 31036 (GOET)	**KX113507**	**KX113483**	**KX113495**
Harpalejeunea uncinata Steph.	Panama, Schäfer-Verwimp 34146 (GOET)	**KX113508**	**KX113484**	**KX113496**
Lejeunea acuta Mitt.	Kenya, Chuah-Petiot Mb 22 (JE)	KF556384	KF556134	KF555917
Lejeunea adpressa Nees	Dominican Republic, Pócs & Pócs 03156/F (EGR)	KF556385	KF556135	KF555918
Lejeunea amaniensis E.W.Jones	Kenya, Malombe & Chituyi 5006/Si.1aS5 (EGR)	KF556392	KF556143	KF556603
Lejeunea anisophylla Mont.	Kingdom of Tonga, Schäfer 18341 (GOET)	KF556394	KF556145	KF555926
Lejeunea asperrima Spruce	Panama, Schäfer-Verwimp & Verwimp 30817 (GOET)	KF556402	KF556157	KF555935
Lejeunea bermudiana (A.Evans) R.M.Schust.	USA, Shaw 14939 (DUKE)	KF556403	KF556158	KF555936
Lejeunea boryana Mont.	French Guiana, Holz FG 00-0103 (GOET)	KF556405	KF556159	KF555938
Lejeunea cancellata Nees & Mont. ex Mont.	Costa Rica, Schäfer-Verwimp & Holz SV/H-0507/C (GOET)	KF556409	KF556164	KF555942
Lejeunea catinulifera Spruce	Ecuador, Wilson et al. 04-01 (GOET)	DQ983687	DQ987432	DQ987328
Lejeunea cavifolia (Ehrh.) Lindb.	Germany, Heinrichs 3695 (GOET)	AY548102	DQ238581	DQ987259
Lejeunea cerina (Lehm. & Lindenb.) Gottsche, Lindenb. & Nees	Costa Rica, Schäfer-Verwimp & Holz SV/H-0471 (GOET)	KF556425	KF556180	KF555955
Lejeunea cocoes Mitt.	Indonesia, Schäfer-Verwimp & Verwimp 21050 (GOET)	KF556430	KF556186	KF555961
Lejeunea controversa Gottsche	French Guiana, Hartmann et al. 04-033 (GOET)	KF556432	KF556189	KF555964

Lejeunea debilis (Lehm. & Lindenb.) Nees & Mont.	Costa Rica, Schluder 7 (HJU)	KF556437	KF556197	KF555969
Lejeunea deplanata Nees	Ecuador, Schäfer-Verwimp et al. 24502/C (GOET)	KF556439	KF556199	KF555971
Lejeunea discreta Lindenb.	Australia, Thiers & Halling 2219 (L)	KF556444	KF556206	KF555977
Lejeunea drehwaldii Heinrichs & Schäf.-Verw.	Peru, Drehwald 4384 (JE)	KF556445	KF556207	KF555978
Lejeunea eckloniana Lindenb.	Madeira, Stech 04-271 (L)	KF556446	KF556210	KF555982
Lejeunea exilis (Reinw., Blume & Nees) Grolle	Indonesia, Schäfer-Verwimp & Verwimp 25231 (GOET)	KF556449	KF556213	KF555985
Lejeunea flava (Sw.) Nees	Dominican Republic, Schäfer-Verwimp & Verwimp 26855/B (GOET)	KF556479	KF556243	KF556009
Lejeunea grossitexta (Steph.) M.E.Reiner & Goda	Panama, Schäfer-Verwimp & Verwimp 31000 (GOET)	KF556491	KF556256	KF556020
Lejeunea holtii Spruce	Madeira, Drehwald & Drehwald 3719 (GOET)	KF556492	KF556258	KF556022
Lejeunea isocalycina (Nees) Spruce	Brazil, Costa & Gradstein 3720 (GOET)	KF556496	KF556262	KF556027
Lejeunea japonica Mitt.	Japan, Mizutani 15618 (L)	KF556499	KF556265	KF556030
Lejeunea laeta (Lehm. & Lindenb.) Gottsche, Lindenb. & Nees	Ecuador, Schäfer-Verwimp et al. 24412 (GOET)	KF556501	KF556267	KF556032
Lejeunea laetevirens Nees & Mont.	Dominican Republic, Schäfer-Verwimp & Verwimp 27079 (GOET)	KF556508	KF556275	KF556037
Lejeunea lamacerina (Steph.) Schiffn.	Azores, Schäfer-Verwimp & Verwimp 29394 (GOET)	KF556510	KF556279	KF556041
Lejeunea lomana E.W.Jones	Réunion, Pócs 08064/L (EGR)	KF556388	KF556138	KF555921
Lejeunea micholitzii Mizut.	Fiji Islands, Pócs & Pócs 03288/DC (EGR)	KF556517	KF556288	KF556049
Lejeunea microloba Taylor	Fiji Islands, Pócs 08013/Y (EGR)	KF556520	KF556291	KF556051
Lejeunea mimula Hürl.	Fiji Islands, Pócs 08034/E (EGR)	KF556523	KF556295	KF556052
Lejeunea minutiloba A.Evans	Chile, Easter Island, Ireland & Bellolio 30138 (JE)	KF556525	KF556297	KF556054
Lejeunea monimiae (Steph.) Steph.	Ecuador, Schäfer-Verwimp & Preussing 23226/A (GOET)	KF556526	KF556298	KF556055
Lejeunea multidentata M.E.Reiner & Mustelier	Dominican Republic, Pócs & Pócs 03157/A (EGR)	KF556528	KF556300	KF556057
Lejeunea obtusangula Spruce	Bolivia, Krömer 869 (LPB)	KF556532	KF556307	KF556063
Lejeunea oligoclada Spruce	Brazil, Schäfer-Verwimp & Verwimp 13590 (GOET)	KF556533	KF556308	KF556064

Lejeunea pallescens Mitt.	Ecuador, Schäfer-Verwimp & Nebel 32731 (GOET)	KF556540	-----------	KF556069
Lejeunea parva (S.Hatt.) Mizut.	Japan, Mizutani 15293 (L)	KF556542	KF556318	KF556072
Lejeunea phyllobola Nees & Mont.	Ecuador, Noeske et al. 204 (GOET)	KF556600	KF556322	KF556076
Lejeunea pterigonia (Lehm. & Lindenb.) Mont.	Bolivia, Churchill et al. 21851 (MO)	KF556546	KF556325	KF556078
Lejeunea puiggariana Steph.	Dominican Republic, Schäfer-Verwimp & Verwimp 27016/A (GOET)	KF556550	KF556329	KF556082
Lejeunea pulchriflora (Pearson) G.E.Lee, Bechteler, Pócs, Schäf.-Verw. & Heinrichs	Tanzania, Pócs 89126/M (EGR)	KT897948	KT897953	KT897943
Lejeunea pulverulenta (Gottsche ex Steph.) M.E.Reiner	Bolivia, Reiner-Drehwald & Drehwald 4517 (GOET)	KF556552	KF556331	KF556084
Lejeunea ramosissima Steph.	São Tomé and Príncipe, Shevock 34348A (EGR)	KF556554	KF556333	KF556086
Lejeunea ramulosa Spruce	Costa Rica, Schäfer-Verwimp & Holz SV/H-0229/A (GOET)	KF556555	KF556335	KF556088
Lejeunea reflexistipula (Lehm. & Lindenb.) Gottsche, Lindenb. & Nees	Ecuador, Schäfer-Verwimp & Nebel 33162 (GOET)	KF556559	KF556340	KF556092
Lejeunea rotundifolia Mitt.	Costa Rica, Schäfer-Verwimp & Holz SV/H-0378 (GOET)	KF556567	KF556348	KF556099
Lejeunea sordida (Nees) Nees	Fiji Islands, Pócs & Pócs 03300/AP (EGR)	KF556570	KF556351	KF556102
Lejeunea subspathulata Spruce	Dominica, Gradstein 6643 (GOET)	KF556585	KF556367	KF556119
Lejeunea tabularis (Spreng.) Gottsche, Lindenb. & Nees	Kenya, Pócs & Pócs 04026/X (EGR)	KF556587	KF556369	KF556121
Lejeunea tapajosensis Spruce	Ecuador, Nöske et al. 204 (GOET)	KF556589	KF556371	KF556122
Lejeunea topoensis Gradst. & M.E.Reiner	Ecuador, Gradstein & Jost 10063 (GOET)	DQ983712	DQ987416	DQ987312
Lejeunea trinitensis Lindenb. & Gottsche	Brazil, Vital 10.168 (JE)	KF556594	KF556376	KF556127
Lejeunea tuberculosa Steph.	São Tomé and Príncipe, Pócs & Pócs 34690 (EGR)	KF556595	KF556377	KF556128
Lejeunea umbilicata (Nees) Nees, Lindenb. & Gottsche	Indonesia, Schäfer-Verwimp & Verwimp 16954 (GOET)	KF556597	KF556379	KF556130
Lejeunea sp. I	Ecuador, Schäfer-Verwimp & Preussing 23533 (GOET)	KF556536	KF556312	KF556066
Lejeunea sp. 2	Vietnam, Pócs 02105/J (EGR)	KF556529	KF556303	KF556629
Lepidolejeunea cordifissa (Taylor) M.E.Reiner	Ecuador, Schäfer-Verwimp & Nebel 33219 (M)	KP635307	KP635333	KP635278
Lepidolejeunea cuspidata (Gottsche) Heinrichs & Schäf.-Verw.	Ecuador, Schäfer-Verwimp & Nebel 32123/C (JE)	KP635320	KP635345	KP635291

Lepidolejeunea delessertii (Nees & Mont.) Grolle	Réunion, Schäfer-Verwimp & Verwimp 20355/B (M)	KF039819	KF039853	KF039781
Lepidolejeunea eluta (Nees) R.M.Schust.	Bolivia, Acebey & Krömer 855 (GOET)	KP635328	KP635353	KP635301
Lepidolejeunea involuta (Gottsche) Grolle	Guadeloupe, Schäfer-Verwimp 22178 (M)	KP635312	KP635337	KP635283
Microlejeunea africana Steph.	São Tomé and Príncipe, Shevock 34576B (GOET)	KC313151	KC313191	KC313113
Microlejeunea ankasica E.W.Jones	Príncipe Island, Shevock 42048 (EGR)	**KX113509**	**KX113485**	**KX113497**
Microlejeunea bullata (Taylor) Steph.	Brazil, Peralta et al. 14921 (SP)	**KX113510**	**KX113486**	**KX113498**
Microlejeunea capillaris (Gottsche) Steph.	Costa Rica, Schäfer-Verwimp & Holz SV/H-0489/B (JE)	KC313152	KC313192	KC313114
Microlejeunea colombiana Bischl.	Dominican Republic, Schäfer-Verwimp & Verwimp 26614/A (JE)	KC313153	KC313193	KC313079
Microlejeunea cystifera Herzog	Brazil, Peralta et al. 15764 (SP)	**KX113511**	**KX113487**	**KX113499**
Microlejeunea filicuspis (Steph.) Heinrichs, Schäf.-Verw., Pócs & S.S.Dong	Fiji Islands, Pócs & Pócs 03304/A (EGR)	KC313138	KC313178	KC313100
Microlejeunea fischeri (Tixier) Heinrichs, Schäf.-Verw., Pócs & S.S.Dong	Uganda, Pócs & Lye 97141/T (EGR)	KC313140	KC313180	KC313102
Microlejeunea globosa (Spruce) Steph.	Brazil, Schäfer-Verwimp 34053 (GOET)	**KX113512**	**KX113488**	**KX113500**
Microlejeunea kamerunensis Steph.	Equatorial Guinea, Müller B999/D (EGR)	**KX113513**	**KX113489**	**KX113501**
Microlejeunea latitans (Hook. f. & Taylor) Heinrichs, Schäf.-Verw., Pócs & S.S.Dong	New Zealand, Schäfer-Verwimp & Verwimp 13869 (JE)	KC313146	KC313186	KC313108
Microlejeunea nyandaruensis Pócs	Kenya, Pócs 02031/RB (EGR)	**KX113514**	**KX113490**	**KX113502**
Microlejeunea punctiformis (Taylor) Steph.	Singapore, Pócs & Tan 99203/C (EGR)	**KX113515**	**KX113491**	**KX113503**
Microlejeunea squarrosa (Steph.), Heinrichs, Schäf.-Verw., Pócs & S.S.Dong	Brazil, Schäfer-Verwimp & Verwimp 14638 (GOET)	KC313158	KC313198	KC313119
Microlejeunea ulicina (Taylor) Steph.	La Palma, Schäfer-Verwimp & Verwimp 24800 (GOET)	KC313154	KC313194	KC313115
